# Supplementary material for: Clinical Impact of Preoperative Biliary Drainage in Patients with Ductal Adenocarcinoma of the Pancreatic Head
Source: Diagnostics (Basel). 2023 Mar 28;13(7):1281. doi: 10.3390/diagnostics13071281 (PMC10093239; doi:10.3390/diagnostics13071281)
Supplement: Supplementary file 1 [file diagnostics-13-01281-s001.zip › diagnostics-2210475-supplementary.pdf]

Supplemental Data

Table S1 – Comparison between patients undergoing preoperative biliary drainage (group 1) and those not submitted to this procedure (group 2).

IQR: interquartile range; AST: aspartate aminotransferase; ALT: alanine aminotransferase; AP: alkaline phosphatase; GGT: gamma-glutamyl transferase; BR: bilirubin; CA 19.9: carbohydrate antigen 19.9; CEA: carcinoembryonic antigen; POPF: postoperative pancreatic fistula; CDC: Clavien-Dindo classification; AJCC: American Joint Committee on Cancer; DFS: disease free survival; OS: overall survival

Variable	**Preoperative biliary drainage**		*p*
	**Group 1: Yes** (N=44)	**Group 2: No** (N=84)	
Age (years)			
Median (IQR)	69 (61-74)	69 (58.25-76)	0.980
Min-Max	40-84	41-86	
Gender			
Female (%)	14 (31.8)	36 (42.9)	0.224
Male (%)	30 (68.2)	48 (57.1)	
Preoperative sodium			
Median (IQR)	137 (135.25-140)	137 (134-139)	0.369
Min-Max	129-142	128-144.7	
Preoperative AST			
Median (IQR)	52 (29-83)	108 (45.50-199.50)	<0.001
Min-Max	17-252	9-668	
Preoperative ALT			
Median (IQR)	55 (28.50-110)	140.50 (64.50-264.75)	<0.001
Min-Max	15-259	7-922	
Preoperative FA			
Median (IQR)	233 (124.50-343.50)	434 (256-600.50)	<0.001
Min-Max	59-714	50-2100	
Preoperative GGT			
Median (IQR)	243 (70.50-401.25)	450 (156-954)	0.004
Min-Max	22-1362	10-3001	
Preoperative BR			
Median (IQR)	3.300 (1.600-10.675)	15.85 (3.500-20.700)	0.001
Min-Max	0.3-29.2	0.3-31.7	
Preoperative Albumin			
Median (IQR)	3.600 (3.200-3.850)	3.700 (3.100-4.000)	0.461
Min-Max	2.3-7.3	1.7-41.0	

Preoperative CA 19.9<br>Median (IQR)<br>Min-Max	 163 (32-949)<br>2-23911	 243.5 (36.25-978.75)<br>2-6879	 0.902
Preoperative CEA<br>Median (IQR)<br>Min-Max	 3.25 (1.925-6.700)<br>0.6-105	 3.2 (1.9-4.9)<br>0.6-49	 0.538
Postoperative hospital stay (days)<br>Median (IQR)<br>Min-Max	 13 (10-16)<br>6-36	 13 (9-20)<br>3-85	 0.763
NSQIP- Surgical Risk Calculator<br>Any complications<br>Mean (Min-Max)<br>Surgical site infection<br>Mean (Min-Max)<br>Readmission<br>Mean (Min-Max)<br>Reintervention<br>Median (IQR)<br>Min-Max<br>Death<br>Median(IQR)<br>Min-Max<br>Sepsis<br>Mean (Min-Max)<br>Delayed gastric emptying<br>Mean (Min-Max)	 <br>30.474 (19-42.4)<br><br>18.858 (13.3-24.6)<br><br>16.142 (11.4-20.8)<br><br>5.2 (4.3-6.1)<br>4.1-6.6<br><br>0.8 (0.4-1.7)<br>0.1-6.5<br><br>8.379 (4.7-12.2)<br><br>15.389 (6.4-25.4)	 <br>28.982 (17-36.2)<br><br>17.769 (12.8-21)<br><br>15.331 (8.2-20)<br><br>4.9 (4.3-5.4)<br>3.1-7.3<br><br>0.6 (0.2-1.5)<br>0-3.4<br><br>7.992 (3-13.1)<br><br>15.979 (9.3-25.3)	 <br>0.305<br><br>0.123<br><br>0.295<br><br>0.383<br><br><br>0.440<br><br><br>0.533<br><br>0.610
Operative time (hours)<br>Median (IQR))<br>Min-Max	 7 (6-8.5)<br>4-16.5	 7.75 (6.375-9)<br>3-11	 0.212
Postoperative morbidity<br>Yes (%)<br>No (%)	 36 (81.8)<br>8 (18.2)	 60 (71.4)<br>22 (26.2)	 0.277
Postoperative hemorrhage<br>Yes (%)<br>No (%)	 7 (15.9)<br>37 (84.1)	 9 (10.7)<br>73 (86.9)	 0.428
Intraabdominal abscess<br>Yes (%)<br>No (%)	 6 (13.6)<br>38 (86.4)	 18 (21.4)<br>64 (76.2)	 0.257
Surgical site infection<br>Yes (%)<br>No (%)	 3 (6.8)<br>41 (93.2)	 9 (10.7)<br>73 (86.9)	 0.539
POPF grade B or C			

Yes (%)	7 (16.7)	12 (15.6)	0.878
No (%)	35 (83.3)	65 (84.4)	
Delayed gastric emptying grade B or C			
Yes (%)	13 (29.5)	25 (29.8)	0.976
No (%)	30 (68.2)	57 (67.9)	
Readmission			
Yes (%)	22 (50)	41 (48.8)	1.000
No (%)	22 (50)	41 (48.8)	
CDC ≥ III			
Yes (%)	13 (29.5)	26 (31)	0.802
No (%)	31 (70.5)	56 (66.7)	
Postoperative mortality			
Yes (%)	2 (4.5)	9 (11)	0.223
No (%)	49 (95.5)	73 (89)	
Intraoperative bile culture			
Positive (%)	20 (45.5)	3 (3.6)	<0.001
Negative (%)	12 (27.3)	45 (55.3)	
Multidrug-resistant bacteria			
Yes (%)	9 (45)	1 (33.3)	1.000
No (%)	11 (55)	2 (66.7)	
Perioperative blood transfusion			
Yes (%)	21 (47.7)	24 (28.6)	0.031
No (%)	23 (52.3)	60 (71.4)	
AJCC pT			
pT1-T2 (%)	36 (81.8)	67 (79.8)	0.780
pT3-T4 (%)	8 (18.2)	17 (20.2)	
Size (cm)			
Median (IQR)	2.55 (2.4-3.5)	3.2 (2.675-4)	0.036
Min-Max	1.5-9	0.4-6.5	
AJCC pN			
N0 (%)	8 (18.2)	20 (23.8)	0.464
N1-N2 (%)	36 (81.8)	64 (76.2)	
AJCC stage			
I-II (%)	30 (68.2)	62 (73.8)	0.501
III-IV (%)	14 (31.8)	22 (26.2)	
Margins			
R0 (%)	22 (50)	39 (46.4)	0.701
R1-R2 (%)	22 (50)	45 (53.6)	
Lymphovascular invasion			
Yes (%)	40 (90.9)	63 (75)	0.031
No (%)	4 (9.1)	21 (25)	
Perineural invasion			

Yes (%)	42 (95.5)	74 (88.1)	0.217
No (%)	2 (4.5)	10 (11.9)	
Recurrence			
Yes (%)	26 (66.7)	44 (59.5)	0.453
No (%)	13 (33.3)	30 (40.5)	
Local recurrence			
Yes (%)	13 (50)	16 (36.4)	0.297
No (%)	13 (50)	27 (61.4)	
Pulmonary recurrence			
Yes (%)	15 (57.7)	14 (31.8)	0.048
No (%)	11 (42.3)	28 (63.6)	
Hepatic recurrence			
Yes (%)	15 (57.7)	27 (61.4)	0.587
No (%)	11 (42.3)	15 (34.1)	
Peritoneal recurrence			
Yes (%)	5 (19.2)	9 (20.5)	0.828
No (%)	21 (80.8)	33 (75)	
DFS (months)			
Median (Min-Max)	10 (3-61)	8 (0-25)	0.192
3-year DFS (%)	7.7	0	
5-year DFS (%)	3.8	0	
OS (months)			
Median (Min-Max)	20 (0-120)	18 (0-156)	0.833
3-year OS (%)	30.2	27.8	
5-year OS (%)	18.1	18.5	

Table S2 – Comparison between patients undergoing preoperative biliary drainage by endoscopic retrograde cholangiopancreatography (ERCP) (group 1.1) and by percutaneous transhepatic cholangiography (PTC) (group 1.2).

IQR: interquartile range; AST: aspartate aminotransferase; ALT: alanine aminotransferase; AP: alkaline phosphatase; GGT: gamma-glutamyl transferase; BR: bilirubin; CA 19.9: carbohydrate antigen 19.9; CEA: carcinoembryonic antigen; POPF: postoperative pancreatic fistula; CDC: Clavien-Dindo classification; AJCC: American Joint Committee on Cancer; DFS: disease free survival; OS: overall survival

Variable	**Preoperative biliary drainage - route**		*p*
	**Group 1.1: ERCP** (N=27)	**Group 1.2: CPT** (N=17)	
Age (years)			
Mean (Min-Max)	65.04 (40-83)	71.47 (59-84)	0.034
Gender			
Female (%)	8 (29.6)	6 (35.3)	0.694
Male (%)	19 (70.4)	11 (64.7)	
Preoperative sodium			
Mean (Min-Max)	138.222 (132-142)	135.588 (129-142)	0.01
Preoperative AST			
Median (IQR)	46 (27-72)	60.50 (38.75-108.50)	0.253
Min-Max	19-174	17-252	
Preoperative ALT			
Median (IQR)	54 (26-111)	56 (38.50-117)	0.847
Min-Max	16-259	15-158	
Preoperative FA			
Median (IQR)	203 (118-396)	235 (149-303)	0.819
Min-Max	59-714	59-458	
Preoperative GGT			
Median (IQR)	237 (47-445)	313 (145-369)	0.971
Min-Max	23-1362	22-1200	
Preoperative BR			
Median (IQR)	3 (1.5-4.5)	9 (1.85-11.90)	0.162
Min-Max	0.3-29.2	1.3-22.9	
Preoperative Albumin			
Median (IQR)	3.7 (3.325-3.950)	3.4 (3.1-3.850)	0.482
Min-Max	2.3-4.4	2.3-7.3	
Preoperative CA 19.9			
Median (IQR)	216 (53.0675-571.25)	163 (3.7-2144)	0.751
Min-Max	2.27-2429	2-23911	
Preoperative CEA			

Median (IQR)	3.3 (1.95-5.35)	3.1 (1.5-8.8)	0.885
Min-Max	0.67-20	0.6-3.1	
Drainage time (days)			
Median (IQR)	33 (23-63.50)	22 (15-38)	0.027
Min-Max	6-507	7-108	
Drainage-related morbidity			
Yes (%)	10 (37)	11 (64.7)	0.074
No (%)	17 (63)	6 (35.3)	
Drainage-related morbidity			
Acute cholangitis			
Yes (%)	6 (60)	2 (18.2)	0.080
No (%)	4 (40)	8 (81.8)	
Acute pancreatitis			
Yes (%)	3 (30)	0	0.090
No (%)	7 (70)	11 (100)	
Bleeding			
Yes (%)	0	3 (27.3)	0.214
No (%)	10 (100)	8 (72.7)	
Obstruction			
Yes (%)	2 (20)	0	0.214
No (%)	8 (80)	11 (100)	
Drain mobilization			
Yes (%)	0	6 (54.5)	0.012
No (%)	10 (100)	5 (45.5)	
Postoperative hospital stay (days)			
Median (IQR)	13 (10-21)	11 (8-29)	0.068
Min-Max	6-36	8-29	
NSQIP- Surgical Risk Calculator			
Any complications			
Mean (Min-Max)	28.167 (19-37.5)	34.429 (29.1-42.4)	0.020
Surgical site infection			
Mean (Min-Max)	18.575 (13.3-24.6)	19.343 (14.8-21.7)	0.610
Readmission			
Mean (Min-Max)	15.133 (11.4-19.5)	17.871 (15.4-20.8)	0.020
Reintervention			
Median (IQR)	4.5 (4.225-5.425)	5.8 (5.4-6.2)	0.034
Min-Max	4.1-6.2	4.6-6.6	
Death			
Median (IQR)	0.4 (0.125-1.375)	1.9 (0.9-3.6)	0.005
Min-Max	0.1-1.7	0.8-6.5	
Sepsis			

Mean (Min-Max)	7.517 (4.7-11.5)	9.857 (7.7-12.2)	0.025
Delayed gastric emptying			
Mean (Min-Max)	15.067 (6.4-25.4)	15.943 (12.6-21.5)	0.695
Operative time (hours)			
Median (IQR))	7 (6-8.375)	8 (6-9)	0.531
Min-Max	4-16.5	4-11	
Postoperative morbidity			
Yes (%)	24 (88.9)	12 (70.6)	0.227
No (%)	3 (11.1)	5 (29.4)	
Postoperative hemorrhage			
Yes (%)	5 (18.5)	2 (11.8)	0.689
No (%)	22 (81.5)	15 (88.2)	
Intraabdominal abscess			
Yes (%)	6 (22.2)	0	0.067
No (%)	21 (77.8)	17 (100)	
Surgical site infection			
Yes (%)	3 (11.1)	0	0.272
No (%)	24 (88.9)	17 (100)	
POPF grade B or C			
Yes (%)	5 (20)	2 (11.8)	0.482
No (%)	20 (80)	15 (88.2)	
Delayed gastric emptying grade B or C			
Yes (%)	12 (44.4)	1 (5.9)	0.014
No (%)	15 (55.6)	15 (88.2)	
Readmission			
Yes (%)	14 (51.9)	8 (47.1)	0.757
No (%)	13 (48.1)	9 (52.9)	
CDC ≥ III			
Yes (%)	16 (59.3)	15 (88.2)	0.040
No (%)	11 (40.7)	2 (11.8)	
Postoperative mortality			
Yes (%)	1 (3.7)	1 (5.9)	1.000
No (%)	26 (96.3)	16 (94.1)	
Intraoperative bile culture			
Positive (%)	15 (55.6)	5 (29.4)	0.130
Negative (%)	5 (18.5)	7 (41.2)	
Multidrug-resistant bacteria			
Yes (%)	7 (46.7)	2 (40)	1.000
No (%)	8 (53.3)	3 (60)	
Perioperative blood transfusion			
Yes (%)	13 (48.1)	8 (47.1)	0.944

No (%)	14 (51.9)	9 (52.9)	
AJCC pT			
pT1-T2 (%)	20 (74.1)	16 (94.1)	0.125
pT3-T4 (%)	7 (25.9)	1 (5.9)	
Size (cm)			
Median (IQR)	3 (2.45-3.65)	2.5 (2.3-3.5)	0.307
Min-Max	1.5-9	1.8-4.5	
AJCC pN			
N0 (%)	4 (14.8)	4 (23.5)	0.690
N1-N2 (%)	23 (85.2)	13 (76.5)	
AJCC stage			
I-II (%)	17 (63)	13 (76.5)	0.349
III-IV (%)	10 (37)	4 (23.5)	
Margins			
R0 (%)	13 (48.1)	9 (52.9)	0.757
R1-R2 (%)	14 (51.9)	8 (47.1)	
Lymphovascular invasion			
Yes (%)	24 (88.9)	16 (94.1)	1.000
No (%)	3 (11.1)	1 (5.9)	
Perineural invasion			
Yes (%)	25 (92.6)	17 (100)	0.515
No (%)	2 (7.4)	0	
Recurrence			
Yes (%)	16 (66.7)	10 (66.7)	1.000
No (%)	8 (33.3)	5 (33.3)	
Local recurrence			
Yes (%)	8 (50)	5 (50)	1.000
No (%)	8 (50)	5 (50)	
Pulmonary recurrence			
Yes (%)	11 (68.8)	4 (40)	0.228
No (%)	5 (31.3)	6 (60)	
Hepatic recurrence			
Yes (%)	7 (43.8)	8 (80)	0.109
No (%)	9 (56.3)	2 (20)	
Peritoneal recurrence			
Yes (%)	2 (12.5)	3 (30)	0.340
No (%)	14 (87.5)	7 (70)	
DFS (months)			
Median (Min-Max)	10 (5-61)	5 (3-19)	0.017
3-year DFS (%)	12.5	0	
5-year DFS (%)	6.3	0	
OS (months)			
Median (Min-Max)	24 (0-120)	14 (0-92)	0.258

3-year OS (%)	36.5	18.5	
5-year OS (%)	18.2	18.5	
